# The effects of four hypocaloric diets containing different levels of sucrose or high fructose corn syrup on weight loss and related parameters

**DOI:** 10.1186/1475-2891-11-55

**Published:** 2012-08-06

**Authors:** Joshua Lowndes, Diana Kawiecki, Sabrina Pardo, Von Nguyen, Kathleen J Melanson, Zhiping Yu, James M Rippe

**Affiliations:** 1 Rippe Lifestyle Institute, 215 Celebration Place, Suite 300, Celebration, FL 34747, USA; 2 Rhode Island University, 202 A Ranger Hall, Kingston, RI, 02881, USA

**Keywords:** High fructose corn syrup, Hypocaloric diet, Weight loss, Dietary counseling

## Abstract

**Background:**

The replacement of sucrose with HFCS in food products has been suggested as playing a role in the development of obesity as a public health issue. The objective of this study was to examine the effects of four equally hypocaloric diets containing different levels of sucrose or high fructose corn syrup (HFCS).

**Methods:**

This was a randomized, prospective, double blind trial, with overweight/obese participants measured for body composition and blood chemistry before and after the completion of 12 weeks following a hypocaloric diet. The average caloric deficit achieved on the hypocaloric diets was 309 kcal.

**Results:**

Reductions were observed in all measures of adiposity including body mass, BMI,% body fat, waist circumference and fat mass for all four hypocaloric groups, as well as reductions in the exercise only group for body mass, BMI and waist circumference.

**Conclusions:**

Similar decreases in weight and indices of adiposity are observed when overweight or obese individuals are fed hypocaloric diets containing levels of sucrose or high fructose corn syrup typically consumed by adults in the United States.

## Introduction

During the past 30 years, the consumption of added sugars has increased [1-3]. Although this represents only a small percentage of the overall increase in energy intake, this has caused some investigators to suggest a linkage between added sugars and weight gain and obesity [4-9]. The American Heart Association (AHA) recently released a Scientific Statement recommending significant restrictions on consumption of added sugars, suggesting that daily consumption in adult males and females should not exceed 150 and 100 calories, respectively [10]. These restrictions, which are lower than levels of added sugars currently consumed by 90% of adults, were framed as a potential way to reduce the burden of obesity and cardiovascular disease.

Over the years a variety of potential causes for obesity have been posited, including increased carbohydrate consumption [11] and most recently an increased consumption of high fructose corn syrup (HFCS) [4]. In particular, some studies in animals have linked consumption of added sugars, in general, and HFCS, in particular, with weight gain and obesity [12-14], although these studies have been criticized for delivering amounts of added sugars above those consumed in the human diet. Given the complexity of energy regulation, it is unlikely that one, single component of the diet causes obesity. Nonetheless, many myths persist in this area and are given traction when prestigious scientific organizations such as the American Heart Association (10) recommend restricting one specific component of the diet.

National recommendations for healthful weight loss focus on strategies that include both overall caloric restriction and increased physical activity [15]. However, few individuals actually follow these guidelines by incorporating both dietary restriction and increased physical activity [16]. Multiple studies have shown that equally hypocaloric diets will result in comparable weight loss irrespective of nutrient composition of these diets [17-19]. Whether macronutrient content of the diet effects weight loss, however, remains a topic of debate and controversy [20-23]. It appears that the critical consideration is adherence to whichever hypocaloric diet is employed [14].

Many of the studies suggesting linkages between added sugar and either cardiovascular disease, diabetes, or other metabolic conditions are based on experiments employing a model comparing pure fructose to pure glucose [24-26], neither of which is commonly consumed in the human diet [27], or on epidemiologic studies which establish associations but not cause and effect [7-9,28,29]. Very few prospective data are available exploring the effects of either sucrose or HFCS (the two largest sources of fructose in the diet) and comparing their effects on body weight and body composition.

It has been argued that it is the fructose moiety of both sucrose and HFCS that is particularly worrisome in terms of potential effects on appetite and subsequent weight gain [4,5,29]. This argument posits that differences in hepatic metabolism between fructose and glucose can contribute to increased caloric consumption because of different effects on short term energy regulating hormones. In particular, studies employing a model of 20% or 25% of total calories ingested as pure fructose compared to similar numbers of calories ingested from pure glucose have suggested that differences in responses of insulin, leptin and ghrelin create circumstances where increased caloric consumption might occur following ingestion of fructose, but not glucose [24-26]. In particular, the failure of fructose in these studies to stimulate insulin production, with subsequent leptin production and suppression of ghrelin, suggested a metabolic situation where increased appetite and subsequent weight gain could occur.

It has been argued by some investigators that an increase in sugar consumption may be a contributing factor to increases in overweight and obesity. However, data from the U.S. Agriculture’s Economic Research Service between 1970 and 2008 showed that the increase in sugar intake over the past 4 decades has been only a small percentage of the overall increase in energy intake. Sugars and caloric sweeteners available for consumption increased by an average of 58 calories per day (from 400 calories to 458 calories) [30] whereas total calories available for individuals in the United States increased 515 kilocalories per day from just over 2,100 calories to just under 2,700 calories [30]. Thus, increases in sweeteners represented approximately 11% of the calorie increase for individuals in the American food supply.

Previous research studies in our laboratory and others employing a model comparing sucrose to HFCS did not reveal any differences in short term energy regulating hormones or appetite when comparing the two sugars [31,32]. This is not surprising given the relatively similar composition of sucrose and HFCS. Sucrose is a disachharide containing 50% fructose and 50% glucose. HFCS has two main forms commonly used in the food supply. HFCS-55, the form of HFCS commonly used to sweeten carbonated soft drinks in the United States consists of 55% fructose and 45% glucose. HFCS-42, the common form of HFCS used in baked goods and other products contains 42% fructose and 58% glucose. We elected to include an “active” control group which utilized exercise only (predominantly through walking) since, in our experience, control groups which do not ask participants to make any changes in their daily lives in weight loss studies have often resulted in extremely high rates of dropout due to dissatisfaction with group selection. Furthermore, individuals often believe that exercise will result in weight loss, despite the fact that most studies suggest that exercise alone results in minimum weight loss. Walking exercise was also included in the four milk consuming groups to make the physical activity portion of this study equivalent across all five groups. Furthermore, current recommendations for healthy weight loss typically involve both energy restriction and physical activity, so we wished to incorporate both of these modalities in our research design.

With these considerations as background, the current study was undertaken to explore whether two different amounts of either sucrose or HFCS, when consumed at current population levels (10% or 20% of calories as fructose, representing the 25^th^ and 50^th^ percentile population fructose intake levels, respectively) have any adverse impact on the ability to lose weight or change body composition when consumed as part of mixed nutrient, hypocaloric diets. To our knowledge, this is the first prospective study to examine the effects of added sugars on overweight or obese individuals attempting to lose weight when sugars are consumed at levels typical of the adult population in the context of hypocaloric, energy restricted diets and modest levels of physical activities.

## Methods and procedures

This study was a 12 week, randomized, prospective, double blind trial involving 247 overweight/obese subjects between the ages of 25–60 conducted at two sites in Orlando, Florida. Staff members and subjects were blinded as to whether or not participants in the trial were consuming HFCS or sucrose. Staff members were, however, aware of whether the subjects were consuming 10% or 20% of calories as added sugar since this information was required in order to prescribe the rest of the hypocaloric diet. Subjects were counseled in private counseling rooms in individual sessions to avoid the possibility of subjects talking to subjects in other groups. Both sites were supervised by the same research team and followed identical protocols. We explored the impact of consuming either sucrose or HFCS at the 25^th^ or 50^th^ percent population fructose consumption levels (10% or 20% of total calories) as a component of mixed nutrient, hypocaloric meal plans in a free-living environment. The study was approved for one site by the Western Institutional Review Board and for the other site by the University of Central Florida Institutional Review Board. All subjects signed informed consent forms.

Men and women between the ages of 25–60 years of age with body mass index (BMI) 27.0-35.0 were recruited. Exclusions included current enrollment in any commercial weight loss program, prescription medicines or supplements for weight loss, or a greater than five pound weight change during the past three months. Individuals with a history of orthopedic limitations that would interfere with the ability to meet prescribed exercise, a history of heart problems, a history of major surgery within the last three months, clinically diagnosed eating disorders or any gastrointestinal disorder, dietary restrictions or allergies to any component of the diet or which would limit the ability to adhere to dietary requirements of the study were all excluded. Physical activity was measured utilizing daily physical activity logs which were reviewed on a weekly basis by exercise physiologists or nutritionists. Cigarette smoking or the use of tobacco products, or consumption of greater than 14 alcoholic beverages per week were also excluded.

Interested individuals were initially screened over the phone to determine eligibility based on self reported data. A standardized screening form and phone script were developed to ensure individuals were screened in a consistent manner. Self reported data including height and weight were verified during the initial clinical visit. Fasting blood samples were also obtained to test for glucose, insulin, lipids and C-reactive protein (CRP).

Each subject performed a second screening visit one week later. During this visit, research dietitians assessed participant dietary intake by analyzing a completed three day food record using the Nutrient Data System Research (NDS-R) Software (University of Minnesota, Minneapolis, Minnesota, USA). Body composition was determined by Dual X-Ray Absorptiometry (General Electric i-DXA). This equipment and methodology have been validated extensively by reputable research laboratories over a wide variety of test subjects [33-35]. Total lean mass, percent fat and trunk fat were all determined by DXA Scan. All females were required to have a negative serum pregnancy test prior to DXA testing Repeat measurements of body mass, waist circumference and body composition were performed after the end of 12 weeks. At this time another fasting blood sample was also obtained. All cholesterol samples were sent to a certified, research based laboratory with error rates of less than 1%.

Following completion of the two qualifying visits, individuals were randomly divided into one of five groups. All groups included a fitness walking program. Exercise physiologists counseled all subjects on a weekly basis. All subjects in the four intervention groups were blinded to group assignments. A control group (exercise only) did not change their habitual diets and this group was considered eucaloric. The following group assignments were made. GROUP #1 (HFCS 10%): sweetener at 10% of total calories (25^th^ percentile of U.S. fructose intake) provided from High Fructose Corn Syrup, plus exercise. GROUP #2 (HFCS 20%): 20% of total calories (50^th^ percentile of U.S. fructose intake) provided through HFCS, plus exercise. GROUP #3 (Suc 10%): 10% of total calories provided (25^th^ percentile of U.S. fructose intake) from sucrose, plus exercise. GROUP #4 (Suc 20%): 20% of total calories provided from sucrose, (50^th^ percentile of U.S. fructose intake), plus exercise. GROUP #5 (EO): control group, habitual (eucaloric) diet, plus exercise. All sweeteners were supplied in 1%, low fat milk (Tetra Pak, Denton, Texas).

All four hypocaloric diets (Groups 1–4) were based on individualized calorie levels using the Mifflin-St Jeor calculation for REE (with activity factor) minus 500 kilocalories (2093 KJ). Study personnel supplied HFCS or sucrose products to subjects on a weekly basis in amounts appropriate to their calorie level. The total meal plan for all four hypocaloric groups was based on the American Diabetes Association (ADA) Exchange List and ranged from 50% - 55% carbohydrates, 15%-20% protein, and 25%-30% fat. These dietary plans utilized American Diabetes Association exchange lists similar in fructose content, so that participants in all four intervention groups were prescribed a comparable amount of fructose from sources other than the sugars provided by the interventions.

Subjects in all four hypocaloric groups were carefully counseled by registered dietitians at diet initiation and weekly thereafter. Menu suggestions and recipes were provided to all volunteers. This was intended to reduce boredom with foods included in the diet and provide helpful guidance for subjects. Diet checklists were used by subjects so they could monitor appropriate consumption of all foods and beverages each day. Vigilant attention to portion size and condiments was emphasized. To promote adherence, foods within all meal plans were those foods that were affordable and fit into most people’s lifestyle. At each weekly counseling session, dietitians reviewed dietary checklists with all the subjects to discuss challenges and encourage continued compliance. Participants in the four intervention groups met with registered dietitians every week and dietary intake patterns were reviewed. At weeks six and twelve all participants in the five groups completed a three day food record.

Individuals in the control condition followed their usual, habitual dietary patterns and met with exercise physiologists on a weekly basis to monitor their exercise prescription status.

This was done to minimize the high attrition rates often associated with subjects in control groups that receive no intervention.

The exercise prescription was the same in all five groups and emphasized walking as the preferred form of exercise, however, other forms of exercise were not prohibited. Participants were encouraged to adhere to recommendations for daily physical activity. Duration of each exercise session was progressively increased from 15 minutes three days a week at the start of the study to 45 minutes three days a week at the end of three weeks and remained at 45 minutes three days a week for the duration of the study . Subjects exercised between 60% and 80% of their maximal aerobic power using their predetermined maximal heart rate to regulate exercise intensity. An additional five minutes of warm up and ten minutes of cool down exercise were also included. To minimize overuse injuries, subjects were encouraged to use a variety of exercise modalities (e.g. walking, cycling, etc.). However, walking exercise was recommended as the main form of exercise.

Data were checked for normalcy and analyzed using a two way (time and group assignment) Analysis of Variance with repeated measures. Only data on those who completed the intervention were included in the analysis. Significant time X group assignment interactions were probed by assessing the within-subject change in each of the 5 groups independently. In addition, changes over the course of 12 weeks (week 12 minus baseline) were calculated and between group differences assessed by one way ANOVA. For all analyses the alpha value was set at 0.05. All data were analyzed using SPSS Advanced Statistics V18.

## Result

### Participants

Baseline characteristics of the 162 study finishers can be seen in Table 1. Of the 247 participants enrolled in the study, 162 (Male = 35, Female = 127) completed the 12-week intervention. On average, those who dropped out or who were withdrawn by the investigators for non-compliance were younger than those who finished the 12-weeks (38.3 ± 10.8 vs 42.9 ± 10.3 years, p < 0.05). Lack of compliance with the consumption of the prescribed amount of milk was the primary reason for participant attrition (n = 38 out of 85), but other reasons included participant unwillingness to commit to the time required (n = 21), intolerance to the milk or unwillingness to consume the amount prescribed (n = 15), Moved out of town (n = 4), pregnancy (n = 3) and general dissatisfaction with the study (n = 4). Drop-out rates were similar across all five groups (Table 1).

**Table 1:** Baseline characteristics on participants (n = 162) who completed the intervention

	**Entire population n = 162**	**10% HFCS n = 36**	**20% HFCS n = 24**	**10% Sucrose n = 29**	**20% Sucrose n = 33**	**EO n = 40**
Age (years	42.8 ± 10.2	40.7 ± 10.3	41.7 ± 11.3	41.7 ± 11.2	42.9 ± 11.2	41.4 ± 10.2
Body Mass (kg)	87.2 ± 12.5	88.9 ± 12.3	89.4 ± 12.8	87.7 ± 14.2	89.1 ± 15.1	86.5 ± 12.7
BMI	31.9 ± 3.3	32.0 ± 3.4	32.2 ± 3.1	31.6 ± 3.7	32.1 ± 3.3	31.8 ± 3.1
Body Fat Percent	43.1 ± 6.5	43.2 ± 6.8	43.5 ± 6.3	44.0 ± 7.2	42.3 ± 5.8	42.4 ± 6.5
Blood Glucose (mmol/L)	4.9 ± 0.4	5.0 ± 0.4	5.0 ± 0.5	5.2 ± 0.7	5.1 ± 0.7	5.1 ± 0.6
Cholesterol (mmol/L)	4.9 ± 1.0	4.8 ± 1.1	4.9 ± 1.0	5.0 ± 1.2	5.0 ± 1.0	5.0 ± 0.8

Note: Attrition rates were not significantly different among the groups (37%, 47%, 40%, 28% and 25% respectively).

### Dietary Intake

Compliance to the sweetened milk in the four intervention groups was very high, with 96.6% of all prescribed servings being consumed over the 12 weeks. Compliance was measured by daily food check lists which were reviewed on a weekly basis with the subject by a research nutritionist. . The dietary intervention prescribed a daily caloric deficit of 500Kcal (2093KJ). Energy intake decreased by 1294KJ (p < 0.001). In the entire cohort, including the exercise group, energy intake decreased by 1231KJ per day (p < 0.001, Table 2). This was consistent across all 5 groups (interaction p > 0.05). Each dietary group also decreased dietary fat while increasing consumption of added sugars. There was also an overall decrease in dietary carbohydrate consumption. Actual sucrose and/or HFCS consumption in the diets could not be measured. Thus, actual sucrose or HFCS intake between the groups is unknown.

**Table 2:** Dietary intake

		**HFCS 10%**	**HFCS 20%**	**Suc 10%**	**Suc 20%**	**EO**	**All**	**Time X group interaction**
Energy Intake (KJ)	**Baseline**	9245 ± 3839	7832 ± 1832	7766 ± 2479	8724 ± 2875	7992 ± 2032	8361 ± 2793	0.099
	**Week 12**	7171 ± 2150	6764 ± 1082	6755 ± 1953	7268 ± 1613	7496 ± 2223	7130 ± 1901***	
Fat (g)	**Baseline**	88.2 ± 48.5	69.4 ± 22.8	70.5 ± 26.5	84.2 ± 35.1	72.3 ±23.0	77.6 ± 34.0	<0.001
	**Week 12**	50.5 ± 22.3***	46.1 ±11.4***	49.9 ± 20.1**	49.0 ± 17.7***	69.8 ± 27.9	54.0 ± 22.9	
Carbohydrates (g)	**Baseline**	269.6 ± 108.8	236.7 ± 74.3	230.6 ± 76.2	249.8 ± 92.4	241.4 ± 67.6	246.9 ± 86.1	0.462
	**Week 12**	241.0 ± 66.6	234.6 ± 41.8	220.1 ± 62.3	250.1 ± 49.6	212.9 ± 74.4	231.4 ± 62.4	
Total Sugar (g)	**Baseline**	117.7 ± 63.2	98.0 ± 53.6	89.2 ± 39.8	101.7 ± 56.8	92.9 ± 42.8	100.5 ± 52.5	<0.001
	**Week 12**	143.9 ± 34.6**	163.2 ± 27.3***	125.2 ± 34.0***	163.3 ± 35.0***	83.8 ± 43.8	133.1 ± 47.0	
Added Sugar (g)	**Baseline**	81.8 ± 56.0	62.0 ± 55.1	63.6 ± 38.5	74.1 ± 50.1	61.3 ± 33.2	69.1 ± 47.2	<0.001
	**Week 12**	67.1 ± 22.5	95.8 ± 20.0*	59.1 ± 26.6	97.8 ± 21.1*	50.3 ± 32.8*	72.2 ± 31.7	

Different than baseline, p < 0.05 *, p < 0.01 **, p < 0.001 ***.

### Body mass and adiposity

**Figure 1:**
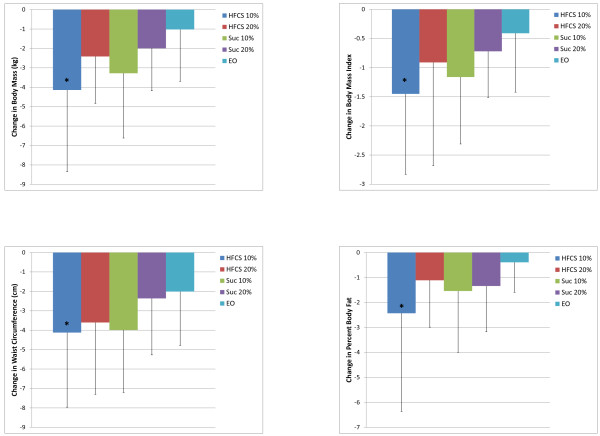
Changes in body mass and measures of adiposity after 12 weeks on a (500Kcal/day) hypercaloric diet containing either 10% or 20% of calories from HFCS.

In the entire cohort, including the non-energy restricted control group (EO), there were reductions in all measures of adiposity (Table 3). Time by group interactions were significant for body mass (p < 0.01), BMI (p < 0.01), waist circumference (p < 0.05) and percent body fat (p < 0.05). Post hoc analysis for within group differences showed that reductions were seen for all measures in all four hypocaloric groups, and also for EO in body mass and BMI (both p < 0.05) and waist circumference (p < 0.001). In all cases the change from baseline to post testing was greater for the HFCS10% than for EO, but in no cases were there any significant difference among the four hypocaloric (Figure 1).

**Table 3:** Changes in body mass and measures of adiposity

		**Baseline**	**Week 12**	**Time X group interaction p**
Body Mass (kg)	HFCS 10%	89.39 ± 11.92	85.24 ± 11.48***	0.003
	HFCS 20%	87.03 ± 11.73	84.61 ± 12.60*	
	Sucrose 10%	86.55 ± 13.10	83.20 ± 12.52***	
	Sucrose 20%	87.76 ± 13.25	85.77 ± 13.26***	
	EO	86.49 ± 12.69	85.46 ±13.36*	
BMI	HFCS 10%	31.48 ± 3.22	30.03 ± 3.30***	0.006
	HFCS 20%	32.30 ± 3.26	31.39 ± 3.65*	
	Sucrose 10%	31.33 ± 3.71	30.17 ± 3.80***	
	Sucrose 20%	31.90 ± 3.15	31.93 ± 3.44***	
	EO	32.34 ± 3.35	30.94 ± 3.52*	
Waist Circumference (cm)	HFCS 10%	91.88 ± 8.04	87.75 ± 8.21***	0.022
	HFCS 20%	90.00 ± 10.88	86.40 ± 10.42***	
	Sucrose 10%	90.75 ± 7.50	86.76 ± 7.97***	
	Sucrose 20%	92.38 ± 9.47	90.01 ± 10.00***	
	EO	93.54 ± 8.79	91.53 ± 8.59***	
Body Fat%	HFCS 10%	42.09 ± 6.98	39.65 ± 9.40**	0.017
	HFCS 20%	42.93 ± 5.58	41.82 ± 5.94*	
	Sucrose 10%	43.75 ± 7.55	42.21 ± 8.22**	
	Sucrose 20%	42.54 ± 6.27	41.20 ± 6.97***	
	EO	43.40 ± 6.55	43.02 ± 6.55	

Different than baseline, p < 0.05 *, p < 0.01 **, p < 0.001 ***.

### Cholesterol and lipids

Reductions in total cholesterol, triglycerides and LDL were observed in the entire cohort (p < 0.001), but no change was observed in HDL (Table 4). Changes in these measures over the 12 weeks were similar among the groups (time X group interaction p > 0.05).

**Table 4:** Changes in cholesterol and lipids

		**Baseline**	**Week 12**	**Time p**	**Time X group interaction p**
Cholesterol (mmol/L)	HFCS 10%	4.78 ± 1.14	4.44 ± 1.11		0.078
	HFCS 20%	4.95 ± 0.89	4.47 ± 0.76		
	Sucrose 10%	5.14 ± 1.18	4.81 ± 0.98		
	Sucrose 20%	5.01 ± 1.04	4.61 ± 0.98		
	EO	4.82 ± 0.08	4.77 0.96		
	All	4.93 ± 1.01	4.63 ± 0.98***	<0.001	
Triglycerides (mmol/L)	HFCS 10%	1.34 ± 0.56	1.22 ± 0.55		0.806
	HFCS 20%	1.30 ± 0.71	1.07 ± 0.50		
	Sucrose 10%	1.33 ± 0.63	1.08 ± 0.34		
	Sucrose 20%	1.42 ± 0.86	1.28 ± 0.70		
	EO	1.55 ± 0.73	1.38 ± 0.67		
	All	1.40 ± 0.70	1.22 ± 0.58***	<0.001	
HDL (mmol/L)	HFCS 10%	1.30 ± 0.22	1.30 ± 0.27		0.182
	HFCS 20%	1.37 ± 0.34	1.28 ±0.27		
	Sucrose 10%	1.41 ± 0.33	1.38 ± 0.35		
	Sucrose 20%	1.34 ± 0.35	1.29 ± 0.32		
	EO	1.25 ± 0.24	1.28 ± 0.23		
	All	1.33 ± 0.30	1.30 ± 0.28	0.090	
LDL (mmol/L)	HFCS 10%	2.87 ± 0.98	2.61 ± 0.91		0.372
	HFCS 20%	2.99 ± 0.78	2.70 ± 0.66		
	Sucrose 10%	3.12 ± 1.02	2.95 ± 0.93		
	Sucrose 20%	2.94 ± 0.94	2.68 ± 0.85		
	EO	2.87 ± 0.74	2.85 ± 0.89		
	All	2.95 ± 0.89	2.76 ± 0.86***	<0.001	

Different than baseline, p < 0.05 *, p < 0.01 **, p < 0.001 ***.

## Discussion

This double blind, randomized, prospective study compared changes in weight and body composition, as well as risk factors for coronary heart disease, type 2 diabetes and the metabolic syndrome in overweight and obese individuals before and after a twelve week, free living intervention during which low fat (1%) milk was prescribed, sweetened by either sucrose or HFCS to deliver 10% or 20% of calories from the sweetener in the context of hypocaloric, mixed nutrient meal plans. This is the first attempt to examine the impact of prescribing either sucrose or HFCS (10% or 20% of calories) at the 25^th^ and 50^th^ percentile fructose population intake levels as a component of mixed nutrient, hypocaloric meal plans in a free living environment. The major finding of this prospective study is that typical population intake levels of added sugars prescribed at the level to deliver the 25^th^ and 50^th^ percentile population levels of fructose consumption [36] does not prevent weight loss and associated improvements in body composition when prescribed in the context of a well designed and supervised weight loss program (Figure 1).

In the current study, individuals in the four intervention groups who started with normal serum cholesterol achieved reductions in serum cholesterol ranging from 13 to 19 mg/dL which is consistent with the amount of weight loss achieved and is clinically significant.

Initial concern was raised that there might be a unique relationship between obesity and the consumption of HFCS because of the temporal association between increased use of HFCS in the American food supply to the increased prevalence of obesity between 1970 and 2000 [4]. Despite the popularity of this suggestion, there are numerous reasons this hypothesis should be discarded. Firstly, the temporal association between HFCS and obesity ended in 1999, when HFCS use began to diminish [30]. Secondly, numerous countries around the world have a similarly increasing prevalence of overweight and obesity as the United States, but do not use HFCS. Lastly, subsequent research studies have shown there is no difference between HFCS or sucrose in any metabolic parameter measured in human beings including glucose, insulin, leptin, ghrelin, triglycerides, uric acid, appetite or calories consumed at the next meal [31,32,37]. Both the American Medical Association [38] and the American Dietetic Association [39] have issued statements declaring that there is nothing unique about HFCS that leads to obesity. Both of these statements note that all caloric sweeteners contain calories and should be used in moderation. The present data further support the theory that, when consumed at levels up to the 50^th^ percentile for fructose in the context of a hypocaloric diet, neither HFCS nor sucrose impedes weight loss. These data provide further support to the concept that overall caloric consumption rather than one particular component of the diet is most important for achieving weight loss.

Recent concern has been raised that it may be the fructose moiety of both sucrose and HFCS that could potentially contribute to obesity [5,6,29]. This argument is based on research performed showing differences in short term energy regulating hormones when comparing a pure fructose model to a pure glucose model [24-26]. Neither fructose nor glucose alone is available in the ordinary food supply as an isolated or pure substance, and neither is consumed alone in significant amounts. It has also been argued that differences in hepatic metabolism between fructose and glucose may stimulate increased caloric consumption and, therefore, increased risk of weight gain and obesity [40-42].

Some epidemiologic studies have reported an increase in energy intake in various population groups related to increased sugar sweetened beverage consumption [7-9]. However, evidence regarding a potential positive association between sugar sweetened beverage consumption and obesity is inconsistent [43]. Because of the metabolic nature of overweight and obesity and the complexity of the western diet, it is unlikely that a single food or food group is the primary cause. Randomized, clinical feeding trials have shown inconsistent results from testing the effects of added sugar on weight gain. Differences in study instruments and methods, population studied and study design may have contributed to these inconsistent findings.

It should be noted that since the added sugars in this study were delivered in low fat milk, the increased consumption of vitamin D may have contributed to some of the results observed. Indeed, in this study 50% increases in vitamin D occurred as a result of milk consumption. Deficiencies in vitamin D and low serum 25 (OH) D levels have been correlated with impaired glucose tolerance, the metabolic syndrome and diabetes independent of obesity [44]. It should also be noted that vitamin D is essential for the metabolism of insulin and may contribute to reduction in the level of CRP [45]. Furthermore, vitamin D may contribute to LDL reduction. Thus, our reported results on cholesterol parameters must be treated with some caution.

Our data demonstrate that equally hypocaloric diets provoked similar weight changes regardless of type or amount of sugar consumed. This finding is not surprising since our research group and others have previously shown the metabolic equivalency of sucrose and HFCS [31,32]. Strengths of the current study are that it is a double blind, randomized, prospective study with a relatively large sample size which explores normal population consumed levels of fructose as delivered through normally-consumed sweeteners, sucrose and HFCS. Weaknesses are that subjects were only followed for twelve weeks and that children, adolescents and elderly subjects over the age of 60 were excluded. A further potential weakness in the current study is the 35% dropout rate, although this dropout rate is consistent with other trials of comparable size and duration [46,47]. The added amount of exercise in this study (45 minutes of walking or comparable exercise three times a week) may have also contributed to the observed weight loss, although most studies report that weight loss from exercise alone is typically modest [48,49]. It should also be noted that 78% of participants in the intervention groups were female. This may limit the ability of these data to be generalized to the public since some animal data suggests that gender influences response to fructose [50,51] and young women are more resistant to fructose induced hypertriglyceridemia than males and hyperinsulinemic women are more susceptible [52-54]. Furthermore, plasma leptin exhibits sexual dimorphism with higher concentrations in women as androgens have a suppressive effect on leptin secretion [55,56]. These are further gender differences which may impact on the ability to generalize from data generated largely in women. Since sucrose and/or HFCS consumptions in the diets could not be measured, the actual differences in intake of these two sugars remain unknown, which should also be taken into consideration in interpreting these data.

Further studies employing larger numbers of subjects from more diverse population groups, and higher doses approaching 90^th^ percentile fructose intakes (approximately 15% of calories as fructose) of either sucrose or HFCS, with longer duration appear warranted.

Common misunderstandings about HFCS [3] have distorted public perceptions, pressuring food manufacturers to replace HFCS with sucrose and municipal and state legislators to mandate removal of HFCS from school nutrition programs. Our data suggest that such actions are pointless and potentially misleading to consumers, since HFCS and sucrose are nutritionally interchangeable.

In conclusion, similar decreases in weight and indices of adiposity are observed when overweight or obese individuals are subjected to hypocaloric diets with different prescribed levels of sucrose or high fructose corn syrup.

## Competing interests

JM Rippe has received research funding from the Corn Refiners Association for the present study. The other study authors reported no competing interests.

## Authors’ contributions

JL and JMR wrote and prepared the manuscript, DK, SP, VN and ZY performed regular dietary assessments and ensured interventional compliance and carried out daily measurement of study parameters, KJM provided technical and scientific assistance. All authors read and approved the final manuscript.

## Funding

This work was supported by a grant from the Corn Refiners Association.
